# Adolescent SBIRT implementation in pediatric primary care: results from a randomized trial in an integrated health-care delivery system

**DOI:** 10.1186/1940-0640-10-S1-A62

**Published:** 2015-02-20

**Authors:** Stacy Sterling, Andrea Kline-Simon, Constance Weisner, Ashley Jones, Derek Satre, Anna Wong

**Affiliations:** 1 Kaiser Permanente Division of Research, Oakland, California, 94612, USA; 2 University of California San Francisco, San Francisco, California, 94143, USA; 3 The Permanente Medical Group, Kaiser Permanente Division of Research, Oakland, CA, 94612, USA

## Background

Substance misuse by adolescents is associated with significant mortality and morbidity [1-9]. In spite of growing evidence on the effectiveness of Screening, Brief Intervention and Referral to Treatment (SBIRT) for adolescents [10-25], it has not been widely implemented in pediatric health-care settings. We describe implementation findings from a trial of different modalities of SBIRT for adolescents during primary care well-visits.

## Materials and methods

We randomized pediatricians (N = 52) from a general pediatrics clinic in an integrated health-care delivery system to three study arms: a “PCP” arm, where pediatricians were trained to deliver SBIRT; a “BHC” arm, where providers referred adolescents who endorsed alcohol or drug (AOD) use or mood symptoms to a behavioral health clinician for SBIRT; and a usual care (UC) arm, where providers had access to assessment tools in the electronic health record (EHR), and referral resources, but were not trained in SBIRT. We used EHR data to examine screening, problem identification, brief intervention, and referral to treatment rates. Brief interventions could focus on alcohol and other drug (AOD) use, mental health (MH), or both problems.

## Results

During the study period there were 8981 well visits; 73 percent of these received initial screening. Initial screening rates were significantly higher in both intervention arms, compared to the UC arm (p < .05). A higher percentage of patients endorsed mood symptoms in the PCP arm (16.4%, BHC = 12.6%, UC = 13.7%; p < .001); endorsement of AOD symptoms did not significantly differ across arms. Approximately 30 percent of teens in each arm were candidates for further assessment, having endorsed at least one of the five AOD or mood risk behavior questions (ns). The percentage of patients endorsing any mood symptoms, who were further assessed per the established SBIRT protocol, was significantly higher in the BHC arm compared to the PCP arm (p < .001); further assessment per the protocol among those with any AOD symptoms was significantly higher in the PCP arm (p < .001). Among those eligible, 25.8 percent in the BHC arm, 16.5 percent in the PCP arm, and 1.8 percent in the UC arm received a BI (p < .001). The percentage of BIs containing any AOD content was significantly higher in the PCP arm compared to the BHC arm (92.6% vs. 59.1%), and the BHC arm delivered more BIs with any MH content (81.8% vs. 10.3%), both p < .001.

## Conclusions

The two intervention arms demonstrated better implementation of different SBIRT components. Findings illustrate challenges to addressing adolescent behavioral health needs inherent in the different models.
